# Four Polygamous Families with Congenital Birth Defects from Fallujah, Iraq

**DOI:** 10.3390/ijerph8010089

**Published:** 2010-12-31

**Authors:** Samira Alaani, Mozhgan Savabieasfahani, Mohammad Tafash, Paola Manduca

**Affiliations:** 1 Fallujah General Hospital, Althubbadh District, Fallujah, 00964, Iraq; E-Mails: samiraalaani@hotmail.com (S.A.); m_tafash@yahoo.com (M.T.); 2 P.O. Box 7038; Ann Arbor, MI 48107, USA; E-Mail: bahar@umich.edu; 3 Medical College, Al-Anbar University, Fallujah, 00964, Iraq; 4 Laboratory of Genetics, DIBIO, University of Genoa, Genoa 16132, Italy

**Keywords:** Iraq, birth defects, war contaminants, epigenetics

## Abstract

Since 2003, congenital malformations have increased to account for 15% of all births in Fallujah, Iraq. Congenital heart defects have the highest incidence, followed by neural tube defects. Similar birth defects were reported in other populations exposed to war contaminants. While the causes of increased prevalence of birth defects are under investigation, we opted to release this communication to contribute to exploration of these issues. By using a questionnaire, containing residential history and activities that may have led to exposure to war contaminants, retrospective reproductive history of four polygamous Fallujah families were documented. Our findings point to sporadic, untargeted events, with different phenotypes in each family and increased recurrence. The prevalence of familial birth defects after 2003 highlights the relevance of epigenetic mechanisms and offers insights to focus research, with the aim of reducing further damage to people’s health.

## 1. Introduction

In Fallujah (Iraq), birth defects, with prevalence of congenital heart defects (CHD) and neural tube defects (NTD), have reached in 2010 unprecedented numbers, above the World average [1]. Lack of a comprehensive birth registry has made it difficult to make an accurate comparison with the pre war period and to understand modalities and dimension of this unusual occurrence in Fallujah. We thus introduced a protocol allowing for reconstruction of the reproductive history of families with birth defects in Fallujah that allows to see the pattern of their presentation in time. We present here the analysis of four cases of fathers with two lineages of progeny, chosen among more than 50 cases under study. Birth defects presented here are classified according to the primary defect as NTD (neural tube defects), CHD (cardiac birth defects), SK (skeletal defects), even when they have additional phenotypes, or O (others). Modalities of presentation within each family suggest that epigenetic factors may be at the origin of the mechanisms responsible for these defects. The timing of the birth defect occurrences suggests that they may be related to war-associated long-term exposure to contamination.

The epigenetic origin of many birth defects is extensively supported by the existing literature [2]. Neural tube defects are rarely due to traceable genomic changes. The high rate of recurrence of anencephaly in siblings (6.3%) is understood as due to environmental factors and maternal effects. Studies of NTD in mice indicate that anencephaly may be due to multifactorial combinations of hypomorphs and low-penetrance heterozygotes. It is not known how many genes may contribute to anencephaly in humans.

Congenital heart defects have various phenotypes and can be compounded by different cardiac-unrelated features. Approximately 30% of CHD and tetralogy of Fallot (TOF) have been shown to be associated with wide genomic rearrangements. Only in a very small fraction of the cases investigated have cohort studies detected mutations in single genes which are putatively involved (e.g., Holt-Oram syndrome). Environmental factors are implied in the induction of most CHD phenotypes, through epigenetic mechanisms.

In mice, cleft lip is caused by epistatic interactions in the context of genetic maternal effects. Cleft lip and palate inheritance patterns suggest that the causes are combinations of genetic and nongenetic factors. Also in mice, synpolydactyly is linked to Hoxd13 mutations and its manifestation is dependent upon environmental factors.

Tumorigenesis is a multistep process involving mutations and epigenetic changes [2]. Repeated cases of infant leukemia in families are due to genetic predisposition and epigenetic changes that can occur *in-utero* by epigenetic modifiers. Transplacental effector-molecules and epigenetic trans-generational mechanisms are implied in the manifestation of infant leukemia.

Thus, although in teratogenesis and infant leukemia can occur through genomic rearrangements or concurrence of multiple single mutational events, they are most often described and understood to be due to pleiotropic epigenetic changes which affect more than one function, or to a combination of these three mechanisms.

Many known war contaminants have the potential to interfere with normal embryonic and fetal development. The devastating reproductive health effects of dioxins (the major contaminant of Agent Orange) on the Vietnamese people is well known. Data is also accumulating on increased rates of reproductive diseases in veterans of U.S. and U.K. wars during the last 20 years. As environmental effectors, metals are potential good candidates to cause birth defects. Metals are also integral to modern “augmented” and “targeted” weapons [3]. Metals, which are toxicants at relatively low concentrations, are highly persistent in the environment and in the body of exposed individuals, where they accumulate. Metals can disrupt events associated with embryo/fetal development and can act synergistically with other metals and/or with other environmental toxicants to induce phenotypic changes at the level of the cell, and to disrupt tissue homeostasis [4]. Many metals are weak mutagens but strong carcinogens, which implies that metals act more commonly at the epigenetic level leading to changes that are inherited by the progeny of cells.

The analysis of four cases of fathers with two lineages of progeny is presented here. In each case, we discuss what can be deduced from the family’s reproductive history, from the phenotypes of their offspring and from the modality of birth defects’ chronological occurrence. We discuss the compatibility of occurrence of birth defects with the actions of potential effectors, with the demographic of the families, and with the exposure of the parents to war-related events. We have put our evaluation into the context of mechanisms of actions for teratogens. The timing of presentation of birth defects in these families shows that they mostly occurred after 2003.

## 2. Experimental Section

We developed an *ad hoc* questionnaire to collect the retrospective reproductive history of families that had come to Fallujah General Hospital for childbirth and treatment. Questions about history of residence, demographic characteristics and lifestyle habits of the parents, and activities that may have led to their exposure to war-contaminants, were included.

## 3. Results and Discussions

**Figure 1:**
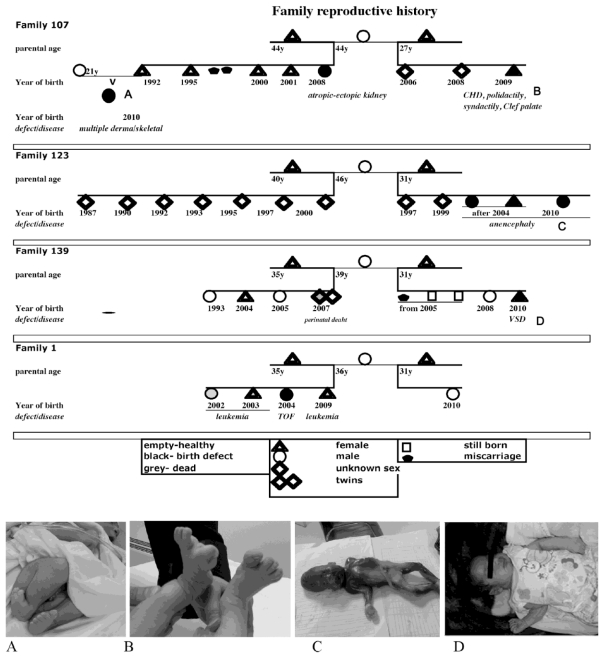
Top panel-family reproductive history is graphically represented. Bottom panel-Photographic record of birth defects: (A) Male child diagnosed with very short webbed neck, rocker bottom feet, malformed thighs, and legs flexed at hips, knees and ankles, retracted penile skin-born full term, August 7, 2010—Family 107—daughter progeny. (B) Female child diagnosed with cleft palate, poly and sindactily of both feet and right hand and congenital heart defect-born at 42 weeks, October 1, 2009—Family 107. (C) Induced abortion (at 22 weeks) of a male fetus with anencephaly, May 31, 2010. (D) Female child, with ventricular septal defect (VDS), born at full term, April 28, 2010—Family 139. Patient consent for publication of the data was obtained from all concerned.

Data from four polygamous families are presented. Mothers were admitted to Fallujah General Hospital between April 2008 and May 2010 for childbirth. Figure 1 summarizes their reproductive histories and illustrates their offspring’s birth defects. Parents’ siblings and their progeny (total n = 40) do not present birth defects or cancer. Figure 1 shows that birth defects occur sporadically with different phenotypes in a family and without obvious suggestion of known genetic contributions from the father or the mother. These represent a few families reported from a greater number of families with birth defects in Fallujah. In May 2010, over 15% of all deliveries (547) in Fallujah General Hospital presented birth defects. During the same period, spontaneous abortions were 14% of assisted pregnancies, premature deliveries (<30 weeks of gestation) were 11%, and there was one stillbirth. These numbers are not significantly different in each of the months of 2010 (manuscript in preparation). Our historical reconstruction of the reproductive lives of these families shows that incidences of birth defects began in 2003 with one exception, namely the infant leukemia case. A composite of genetic and epigenetic factors is understood to cause infant leukemia. The same family also presented a sporadic case of unrelated birth defect, TOF. Table 1 summarizes demographic characteristics and lifestyle habits of the parents of the malformed children born in Fallujah General Hospital (Iraq), between April 2008 and May 2010.

The data shows that each was stably resident, in different areas of the town up to 2009–2010, none of the parents were directly wounded or trapped under rubble and that, among them, only the male parent in Family 1 reported acute symptoms immediately after bombings. There is no obvious relationship to immediately adjacent bombing/burning of their houses or to the activity of cleaning/recovery of injured-dead people or to personal acute symptoms with them having a child with birth defect in the following years. This suggests that the birth defect in these families might not be due directly to acute exposure, but could be associated to their long term exposure and body accumulation of toxicants which are persistent in the environment.

More efficaciously than chromosomal mutations or than multiple single gene mutations, epigenetic changes (alone or associated with other genetic changes) can account for the patterns and diversity of malformation which we see in families 107, 139 and 1. Epigenetic effectors can produce simultaneously different damages and multiple phenotypes, which vary depending on the specific agent(s), and on the timing and level of exposure during embryonic life. This possibility, per se, would fit the data. The novel presentation of sporadic and diverse birth defects, which can be caused uniquely or in composite fashion by epigenetic events, also suggest that agents capable of initiating these changes are still present in the environment and continue to induce novel effects.

Teratogens in the postwar environment include metals and metal alloys which persist in the environment and in the body, and are potential risks to health (genotoxic, fetotoxic and epigenetic mechanisms of action). Metals are involved in regulating genome stability, in X chromosome inactivation, in gene imprinting, and in reprogramming gene expression. They act as metalloestrogens, inhibit DNA repair, alter DNA methylation, change transcriptome and microRNAs production, histone acetylation and methylation; all of which can lead to birth defects, whether translated into mutations or not [4]. As a consequence of internal radiation, some metals can induce sporadic gene mutations or oxidative DNA damage. In the case of depleted uranium (DU) it is unclear whether its radiation-derived mutational effects or its chemical toxic effects are more relevant. DU can induce epigenetic changes that are associated with leukemia via hypomethylation of the DNA. Exposure to teratogens of either father or mothers are potentially effective to induce birth defects at the epigenetic as well as the genetic level. In the cases we report here, the pattern of presentation does not exclude the contribution of either parent: the epigenetic changes are likely to behave as stochastic and not striclty deterministic events, and lack of effects in one of the two families branch with the same father cannot exclude his contribution to the occurrence of birth defects in the other family branch. Nonetheless, pregnant mothers’ exposure to metal contaminants is potentially more relevant to the development of malformations in the case of Family 107, where both wives had deformed babies, but with different phenotypes, and where the daughter of one of the mothers, who herself delivered a child with atrophic and ectopic kidney, had a child with multiple skeletal and dermal abnormalities. There are no known candidate mutations governing both kidney and skeletal/dermal development, and it is likely that independent exposure to effectors in the environment during both pregnancies induced diverse epigenetic effects in the developing offspring. Prenatal exposure best explain, but also paternal exposure could account for, the cases in those families where only the progeny of one spouse presented birth defects which recurred in diverse phenotypes. Cases in point are the still births and ventricular septal defect (VDS) in Family 139 and multiple cases of child leukemia and TOF in Family 1.

Continuing exposure to environmental effectors could also explain the unusually high frequency of recurrence of anencephaly and infant leukemia in a genetically-prone context (Family 123 and Family 1). These phenotypes are indeed known to derive from concomitant genetic predisposition (possibly informing the maternal effect) and from epigenetic effects due to the external environment.

Frequent miscarriages in several women during the last years are also indicative of a general negative (teratogenic) load from the environment. Epidemiological evidence on birth defects which are caused by war contaminants is common in the literature. Hindin *et al.* offered a review of epidemiological studies on the teratogenicity of DU and concludes that human epidemiological evidence is consistent with increased risk of birth defects in the offspring of persons exposed to this war contaminant [5]. Studies in another war contaminant, Agent Orange, also find parental exposure to be associated with an increased risk of birth defects in the offspring [6].

The family history questionnaire which we developed filled serious gaps in a long history of inadequacies of the health system in Iraq. It has allowed us to begin interpretation of the facts. The strong indication of our data, that epigenetic mechanisms are at the root of the recurrence of birth defects in Fallujah, offers the hope to develop therapeutic interventions for severely affected families. Our findings can lead to a deeper understanding of the effects of war contaminants (including metalloestrogens), can help elucidate causes and mechanisms that have culminated in such high rates of birth defects in Fallujah, and can open the way to intervention, both regarding immediate counseling and in terms of therapeutic intervention.

## 4. Conclusions

We conclude that the high prevalence of birth defects in Fallujah is impairing the population’s health and its capacity to care for the surviving children. These defects could be due to environmental contaminants which are known components of modern weaponry. Investigations of metal contaminants, and elucidation of the types and body burden of metals, combined with simultaneous registry of the population’s reproductive history, will allow the identification of families at high risk and will facilitate therapeutic measures to remediate the damages.

## Figures and Tables

**Table 1:** Demographic characteristics, lifestyle habits, history of residence and activities that may have led to exposure to war-contaminants of the parents of malformed children born in Fallujah General Hospital (Iraq), between April 2008 and May 2010.

	Birth defect	Age	Race	Education	Occupation	Smoking/Alcohol		House/OR vicinity bombed	White Phosphorus burns	Rescue/Rubble clearing	Acute poisoning sympthoms	Residence 2003/2010
***Family107***												
Father		**44**	**White**	**High School diploma**	**Day laborer**	**No**	**No**	**No/No**	**No**	**No**	**No**	**Baghdad/2006 Fallujah Alshuhadaa**
First wife	O	**44**	**White**	**Elementary School**	**House wife**	**No**	**No**	**No/No**	**No**	**No**	**No**	**Baghdad/2006 Fallujah Alshuhadaa**
Second wife	CHD	**27**	**White**	**Junior High School**	**House wife**	**No**	**No**	**No/No**	**No**	**No**	**No**	**Baghdad/2006 Fallujah Alshuhadaa**
first female child	SK	**21**	**White**		**House wife**	**No**	**No**	**No/No**	**No**	**No**	**No**	**Baghdad/2006 Fallujah Alshuhadaa**
***Family123***												
Father		**46**	**White**	**Police Academy graduate**	**Police officer**	**No**	**No**					**Fallujah- Daffar**
First wife		**40**	**White**	**Elementary School**	**House wife**	**No**	**No**	**No/Yes2004**	**No**	**No**	**No**	**Fallujah- Daffar**
Second wife	NT-NT- NT	**31**	**White**	**Elementary School**	**House wife**	**No**	**No**	**No/Yes2004**	**No**	**No**	**No**	**Fallujah- Daffar**
***Family139***												
**Father**		**39**	**White**	**High School diploma**	**Day laborer**	**Yes**	**No**	**No/No**	**No**	**Yes**	**No**	**Fallujah City**
First wife	CHD	**35**	**White**	**Elementary School**	**House wife**	**No**	**No**	**No/No**	**No**	**Yes**	**No**	**Fallujah City**
Second wife		**31**	**White**	**High School diploma**	**House wife**	**No**	**No**	**No/No**	**No**	**Yes**	**No**	**Fallujah City**
***Family 1***												
Father	CHD	**36**	**White**	**Military Academy graduate**	**Military officer**	**No**	**No**	**Yes/Yes 2004**	**No**	**Yes**	**Yes**	**Fallujah-Aljumhooreya/2009 Garmah**
First wife		**35**	**White**	**Elementary School**	**House wife**	**No**	**No**	**Yes/Yes 2004**	**No**	**No**	**No**	**Fallujah-Aljumhooreya/2009 Garmah**
Second wife		**31**	**White**	**Teachers College graduate**	**Elementary school teacher**	**No**	**No**	**Yes/Yes 2004**	**No**	**No**	**No**	**Fallujah-Aljumhooreya/2009 Garmah**

Demographics and life style habits of four Fallujah families (**NT** = Neural tube defect; **CHD** = Congenital heart defect; **SK** = Skeletal defect; **O =** Other defect).
